# Oseltamivir- and Amantadine-Resistant Influenza Virus A (H1N1)

**DOI:** 10.3201/eid1601.091304

**Published:** 2010-01

**Authors:** Peter K.C. Cheng, Amanda P.C. To, Tommy W.C. Leung, Peter C.K. Leung, Connie W.C. Lee, Wilina W.L. Lim

**Affiliations:** Department of Health, Hong Kong, People’s Republic of China

**Keywords:** Oseltamivir, amantadine, double resistance, antimicrobial resistance, H1N1, influenza, viruses, expedited, letter

**To the Editor:** We previously reported detection of double resistance to oseltamivir and amantadine of influenza virus A (H1N1) in Hong Kong during the first half of 2008 (*1*). Three different strains of A/Hong Kong/2652/2006-like (clade 2C) viruses that carried the S31N mutation in the matrix (M2) gene associated with amantadine resistance acquired a neuraminidase (NA) gene with CAT→TAT change at position 274 through either reassortment with an oseltamivir-resistant A/Brisbane/59/2007-like (clade 2B) virus or spontaneous H274Y mutation in the NA gene. A clade 2C strain resistant to both oseltamivir and amantadine also was detected in Cambodia in 2007 (*2*).

From July 2008 through December 2008, we continued to monitor antiviral susceptibility of all influenza A (H1N1) viruses in our laboratory, using previously described methods (*1*). Resistance to oseltamivir increased from 16.9% in July to 97.8% in December (Table). Sequencing of the hemagglutinin (HA) gene showed that, beginning in October, A/Brisbane/59/2007-like clade 2B virus had overtaken A/Hong Kong/2652/2006-like clade 2C virus to become the predominating circulating influenza A virus (H1N1) in Hong Kong. Of 916 isolates, 6 (0.7%), isolated from July through September 2008, were resistant to both amantadine and oseltamivir. Genetic analysis showed that 5 were similar to those we described previously, 4 were A/Hong Kong/2652/2006-like clade 2C viruses with spontaneous H274Y mutation in the NA gene, and 1 was a clade 2C virus but acquired a clade 2B NA gene carrying the H274Y mutation. The sixth double-resistant virus was an A/Brisbane/59/2007-like clade 2B virus with a spontaneous S31N mutation in the M2 gene. No epidemiologic link was detectable between these viruses. From October through December 2008, no double-resistant viruses were detected.

**Table:** Prevalence of oseltamivir-resistant and amantadine- and oseltamivir-resistant influenza A virus (H1N1) detected in Hong Kong, with clade designations, July 2008–June 2009

Date detected	No. isolates tested	No. (%) isolates oseltamivir resistant	No. (%) isolates oseltamivir and amantadine resistant	Predominating influenza A virus (H1N1) clade, no. identified/total no. sequenced (%)
2008 Jul	462	78 (16.9)	4 (5.1)	Clade 2C, 104/182 (57.1)
2008 Aug	313	45 (14.4)	1 (2.2)	Clade 2C, 51/95 (53.7)
2008 Sep	61	21 (34.4)	1 (4.8)	Clade 2B, 20/39 (51.3)
2008 Oct	19	13 (68.4)	0	Clade 2B, 13/16 (81.3)
2008 Nov	16	15 (93.8)	0	Clade 2B, 16/16 (100)
2008 Dec	45	44 (97.8)	0	Clade 2B, 41/41 (100)
2009 Jan	327	313 (95.7)	0	Clade 2B, 90/104 (86.5)
2009 Feb	769	755 (98.2)	0	Clade 2B, 138/153 (90.2)
2009 Mar	279	279 (100)	0	Clade 2B, 61/61 (100)
2009 Apr	63	63 (100)	2 (3.2)	Clade 2B, 18/18 (100)
2009 May	22	22 (100)	2 (9.1)	Clade 2B, 7/7 (100)
2009 Jun	77	77 (100)	46 (59.7)	Clade 2B, 55/55 (100)

From January through June 2009, A/Brisbane/59/2007-like clade 2B virus continued to be the predominating strain. Of the total 1,537 influenza virus A (H1N1) isolates tested during the period, 1,509 (98.2%) were resistant to oseltamivir. Of the 1,509 oseltamivir-resistant isolates tested from April through June 2009, 50 (3.3%) also were resistant to amantadine (Table). Nucleotide sequencing of the HA, NA, and M2 genes was performed on all 50 oseltamivir- and amantadine-resistant viruses. All were A/Brisbane/59/2007-like clade 2B viruses that had acquired an M2 gene carrying the S31N mutation by reassortment with an amantadine-resistant A/Hong Kong/2652/2006-like clade 2C virus. Nucleotide sequencing of the other 5 internal genes (nonstructural, nucleoprotein, polymerase acidic, polymerase basic 1, and polymerase basic 2 proteins) was performed on 2 double-resistant strains isolated in April and on 3 isolated in June. Sequence comparison showed that 1 virus in April, in addition to acquiring an M2 gene, acquired a nonstructural protein gene from an A/Hong Kong/2652/2006-like clade 2C virus. All the viruses were susceptible to zanamivir and were not associated with unusual severity of disease.

Along with pandemic (H1N1) 2009, seasonal influenza viruses continue to circulate in Hong Kong (*3*). An alarming proportion of the circulating seasonal influenza virus A (H1N1) became resistant to both oseltamivir and amantadine in a short span of 1 month. Oseltamivir-resistant A/Brisbane/59/2007-like clade 2B virus that had reassorted with A/Hong Kong/2652/2006-like clade 2C virus had apparently spread in the community and to other regions of the world. The possibility of reassortment with pandemic (H1N1) 2009 virus is a major concern. Resistance to oseltamivir of pandemic (H1N1) 2009 virus will compromise its use in treatment and render the billion-dose stockpile useless. Although the recently detected oseltamivir-resistant pandemic (H1N1) 2009 virus in Hong Kong was not a reassortant virus (*4*,*5*), we will continue to closely monitor antiviral drug resistance among circulating viruses, including pandemic (H1N1) 2009 virus and seasonal influenza virus A (H1N1), as well as influenza A (H3N2) viruses, to track how antiviral resistance evolves.
